# Remaining Useful Life Prediction of Bearings via Semi-Supervised Transfer Learning Based on an Anti-Self-Healing Health Indicator

**DOI:** 10.3390/s25123662

**Published:** 2025-06-11

**Authors:** Jung-Woo Kim, Kyoung-Su Park

**Affiliations:** Department of Mechanical Engineering, Gachon University, 1342 Seongnamdaero, Sujeong-gu, Seongnam-si 461-701, Republic of Korea; brucekim1212@naver.com

**Keywords:** semi-supervised transfer learning, remaining useful life, bearing, anti-self-healing, attention mechanism

## Abstract

Remaining useful life (RUL) estimation of a bearing is a methodology to monitor rolling bearings for a system’s performance and reliability. It predicts the exact residual time without operational interruptions until complete bearing failure by training a deep learning model to predict the remaining time of working using extracted signal features. Extracting features is one of the most important subjects since its quality directly influences the performance of predicting RUL. Features should gradually and consistently increase over time and capture sudden deterioration within normalized specific thresholds. However, recent studies have not addressed feature extraction methods that consider all of these aspects. Moreover, some bearings exhibit a “self-healing” phenomenon, in which bearing conditions appear to temporarily improve, and this complicates the accurate representation of consistent performance degradation. However, very few studies have properly addressed this issue. Meanwhile, transfer learning is frequently used when training the RUL deep learning model because there is a lack of data for run-to-failure experiments. Most RUL estimation methodologies pre-train and apply deep learning models with supervised learning. But supervised transfer learning supposes that researchers already have access to end-of-life (EOL) data—often unavailable in industrial settings—limiting their practicality. To address these challenges, this paper proposes a novel semi-supervised transfer learning methodology that integrates an anti-self-healing health indicator (ASH-HI) with a transformer-based architecture. ASH-HI is a health indicator that quantifies the power spectrum density (PSD) difference between normal and abnormal states using skewness-based parameter selection, eliminating the need for manual parameter tuning. Also, it overcomes the self-healing problem by measuring the difference not only between normal and abnormal states but also between “correction” and abnormal states. Also, this paper presents a new semi-supervised transfer learning method without EOL information. The proposed methodology is validated using the PHM 2012, NASA IMS, and an experimental setup. This study is the first to attempt transfer learning using more than three datasets simultaneously, resulting in significantly improved performance.

## 1. Introduction

Rolling bearings are widely used as components in mechanical systems designed to support the load of rotating systems while reducing friction between connected parts to ensure smooth rotational motion. Predicting the health status and remaining useful life (RUL) of such bearings directly impacts the overall performance of the system [1,2,3]. In general, minor defects in bearings gradually progress because of continuous operation, which significantly impacts the RUL of the bearing. Therefore, the development of accurate RUL prediction techniques is essential for enhancing the overall performance of mechanical systems, including bearings, while reducing maintenance, repair, and operation (MRO) costs [4]. To monitor bearing health status without halting mechanical system operations, conventional methods have focused on analyzing the statistical or physical characteristics (fault frequencies) of vibration signals [5,6,7]. However, these conventional methods typically achieve an accuracy of less than 90%, and in most cases, defects are detected only after significant system degradation has occurred—that is, when the root mean square (RMS) magnitude of vibrations (or acoustics) has substantially increased [8,9]. To overcome these limitations, recent advancements in deep learning and machine learning algorithms have enabled the direct quantification of the remaining operational time of a system before failure, leading to the development of advanced RUL prediction methodologies [10]. These innovations have significantly improved detection performance to over 95%, as well as MRO performance in mechanical systems, including bearings [11,12,13]. Deep learning techniques for predicting the remaining useful life (RUL) of bearings can be broadly categorized into two criteria: whether they employ transfer learning or not and whether they are based on supervised or un-supervised learning. Transfer learning is applied in real industrial systems because it is often challenging to obtain sufficient data on defective products due to well-optimized mass production processes, or acquiring such data requires a significant time investment. To address this issue, pre-trained models are developed using laboratory-level test setups or open datasets and then applied to real systems, allowing optimal performance to be achieved with minimal production data by adjusting the weighting matrix to fit the target system. Most transfer learning methods rely on supervised learning, using datasets with well-labeled characteristics at each lifespan stage. This approach aims to enhance target domain performance by training in both a data-rich source domain and a data-scarce target domain [14,15,16]. However, as previously mentioned, supervised learning requires knowledge of the bearing’s complete failure point or end-of-life (EOL) information, which is practically unattainable in real industrial environments. Consequently, this limitation results in poor model generalization [17]. Wang et al. [18] proposed an objective function utilizing a temporal convolutional network to ensure that learned representations exhibit a consistent distribution, addressing the issue of datasets without RUL labels. However, this method assumes that the source and target domains share the same distribution, leading to performance degradation when this assumption does not hold. Ruonan et al. [19] trained the RUL prediction main model and the fault diagnosis classifier simultaneously during the pre-training stage and adapted the model to the target domain by training only the fault diagnosis classifier during the transfer learning stage. Similarly, Tao et al. [20] enhanced RUL prediction performance through domain adaptation by using a classifier that distinguishes between the two domains (source and target), encouraging the model to learn similar representations for the source and target domains. By using one-hot encoding for labeling, it is assumed that the fault modes or the two domains are discrete and completely unrelated. However, it is unreasonable to regard them as entirely distinct datasets, which presents a clear limitation. Cheng et al. [21] introduced a dual-transferable attention mechanism-based deep learning framework to facilitate learning similar representations between source and target domains. To further tackle the challenge of predicting RUL without labeled datasets, Berghout et al. [22] proposed a transfer learning methodology without RUL labels, defining the concept of a health indicator and using it for regression and health state classification. However, this approach also relies on preprocessing methods that require EOL information, limiting its practical application. Additionally, even when EOL information is available, most bearing vibration data measurements show a physical phenomenon called “self-healing” [23,24], where the RMS value of the signal initially increases near the end of the RUL phase, momentarily decreases, and then rises again. This can mislead learning models, causing them to incorrectly interpret the self-healing phenomenon as an actual extension of the degradation process, thereby introducing significant errors in RUL prediction [23]. The inclusion of self-healing effects in datasets leads to inconsistent variations in health indicators (HIs) [23], negatively impacting model training and potentially causing safety hazards or downtime in real industrial applications. Therefore, it is crucial to address the degradation performance deterioration caused by self-healing phenomena and develop an RUL prediction system that can be applied to real industrial systems without requiring labels or EOL information.

In this study, we propose a transfer learning-based RUL prediction algorithm that significantly enhances the robustness and accuracy of deep learning models by leveraging advanced HI learning and an anti-self-healing (ASH) technology instead of RMS signals. To minimize the impact of self-healing phenomena, an advanced ASH-HI, which measures differences between initial and current frequency spectra, is introduced. Additionally, a skewness-based parameter selection algorithm for automated preprocessing parameter adjustments, providing broader applicability, is applied. For the baseline model, the transformer-based deep learning architecture learns ASH-HI corresponding to the actual system RUL instead of the RMS vibration data. The novelty of its architecture is that it presents a new paradigm of adjusting a pre-trained model to the target bearing with a regression objective function. This helps the model to recognize the source and target domains as completely discrete datasets.

This architecture was validated through application in an in situ experimental setup to ensure reliability. To verify the proposed methodology, various datasets were validated in experiments in Section 2. Section 3 explains the calculation methods for frequency-based health indicators and self-healing prevention factors. Section 4 presents the Transformer-based deep learning model and transfer learning methodology. Section 5 demonstrates the superiority of our approach using benchmark datasets and large-scale bearing experiments, evaluated via the mean squared error (MSE) metric for HI and RUL prediction. Finally, Section 6 discusses conclusions and research findings.

## 2. Datasets

We utilized both benchmark datasets and experiment datasets for verification, with the aim of confirming the generalizability of performance. The two datasets were obtained from systems operating under different conditions, including various bearing sizes, loads, and revolutions per minute (rpm), as shown in Table 1.

### 2.1. Benchmark Datasets

The PHM 2012 [25] and the NASA IMS [26] datasets include data from numerous bearings under different distinct operational conditions: RPM, load, and bearing size. Both datasets utilize harsh experimental conditions to conduct run-to-failure tests in a short time, with the NASA IMS dataset typically having a longer lifespan on average. In the case of the PHM 2012 dataset, only the training dataset includes data up to the EOL, and only the bearing data were utilized because the information on the first predicting time (FPT) [27], which will be explained later, was needed.

**Table 1:** Benchmark datasets’ operating conditions.

Dataset	RPM	Load	Running Time	Bearing Size	Sampling Frequency
PHM 2012	1800 RPM	4000 N	7.7 h	25.6 mm	25.6 kHz
PHM 2012	1800 RPM	4000 N	2.5 h	25.6 mm	25.6 kHz
PHM 2012	1650 RPM	4200 N	2.5 h	25.6 mm	25.6 kHz
PHM 2012	1650 RPM	4200 N	2.1 h	25.6 mm	25.6 kHz
PHM 2012	1500 RPM	5000 N	1.5 h	25.6 mm	25.6 kHz
PHM 2012	1500 RPM	5000 N	4.5 h	25.6 mm	25.6 kHz
NASA IMS	2000 RPM	26,000 N	34 days	71.5 mm	20 kHz
NASA IMS	2000 RPM	26,000 N	34 days	71.5 mm	20 kHz
NASA IMS	2000 RPM	26,000 N	7 days	71.5 mm	20 kHz

### 2.2. Experimental Setup

To verify our proposed algorithm, we used an in-house bearing RUL test setup with slightly larger-sized and heavy-duty bearings, specifically focusing on single-row cylindrical slewing bearings.

#### 2.2.1. Hardware Construction

**Figure 1:**
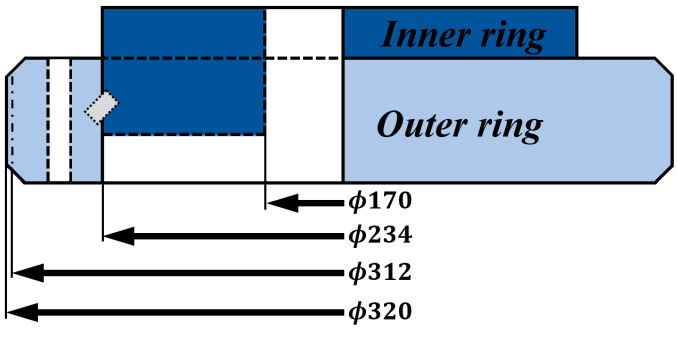
Bearing description.

**Figure 2:**
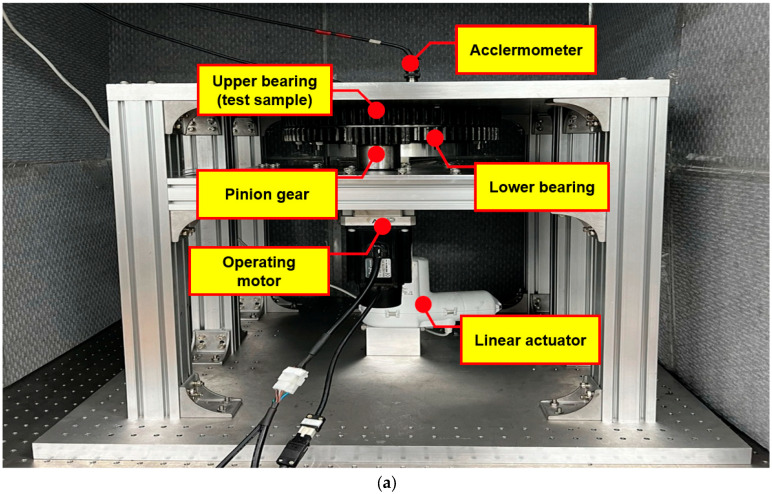

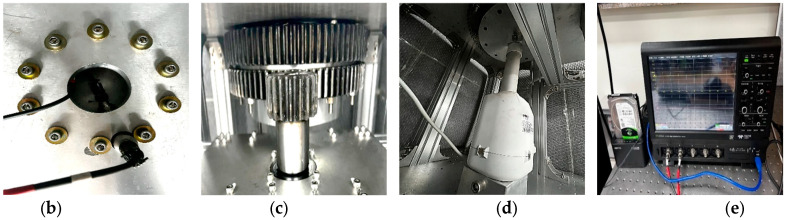
Slewing bearing’s RUL experimental setup: (**a**) setup, (**b**) accelerometers, (**c**) two bearings, (**d**) linear actuator, and (**e**) oscilloscope.

A cylindrical roller bearing with an outer diameter of 320 mm, an inner diameter of 280 mm, a gear pitch diameter of 312 mm, a bearing pitch diameter of 234 mm, a contact angle of 45°, and 78 teeth and 63 cylindrical rollers, each with a diameter of 12 mm, was used, as shown in Table 2 and Figure 1. The designed aluminum frame was fixed to a vibration isolation table, and a motor was mounted at the bottom of the frame to rotate the bearing while isolating external vibrations. As the motor (Higen, FMA-CN06), connected to an 80 mm outer diameter pinion gear with a pitch circle diameter of 78mm and 18 teeth, rotated, the slewing bearing was driven by the gear ratio of 1:4.33. The system was implemented as illustrated in Figure 2. At the center of the setup, there is a linear actuator (LINAK, LA34) to give a vertical load to the bearings. In a linear actuator, a vertical load is applied to perform an accelerated test due to the characteristics of RUL. Since evaluating a bearing RUL under normal conditions takes a considerable amount of time, a test was conducted by applying a 10,000 N load using a linear actuator to enable a faster RUL assessment. Additionally, to allow free rotation of the test sample, the lower bearing and the upper bearing were connected, as shown in Figure 2, to ensure smooth rotation. The system was designed and constructed accordingly. Furthermore, to enable health monitoring during the rotation of the roller bearing, accelerometers (PCB Piezoelectronics, 626B02), capable of measuring low-frequency signals (0.1 Hz~), were installed in both the radial and the axial directions. This setup allowed for the correlation between vibration characteristics and the RUL of the bearing during operation to be derived.

**Table 2:** Geometrical specifications of the slewing bearing.

Outer Diameter	Inner Diameter	Gear Pitch Diameter	Roller Diameter	Bearing Pitch Diameter	Contact Angle	# of Rollers	# of Teeth
320 mm	280 mm	312 mm	12 mm	234 mm	45°	61	78

#### 2.2.2. Data Acquisition

Since the proposed experiment’s bearings were designed for low RPM conditions, and because the measured signals have DC offset signals, we analyzed the signal using an oscilloscope that is capable of AC coupling analysis and also saving the data to a hard drive. The power frequencies were 17 Hz at 30 RPM, and strong energies were detected at harmonics of the power frequency, as observed in [28,29,30,31]. The expression of power frequency can be calculated as in [32].

(1) $$f_{power}=\frac{{RPM}_{rotate}\times Pairs\;of\;poles}{60}$$

We collected 5433 samples at a sampling rate of 2000 Hz over 50 s during a continuous operation after initiating the bearing. The collection was stopped once excessive force disabled its rotation. Slewing bearings rotate at low speeds, which typically extends their expected lifespans, making it difficult to quickly obtain EOL information under normal usage conditions. Therefore, we conducted accelerated testing by operating the main bearing without lubrication at the maximum recommended speed of 30 RPM. This method imposed extremely harsh conditions on the bearing, causing it to fail within just 72 h.

## 3. Anti-Self-Healing Health Indicator

We introduced the ASH-HI, a novel normalized HI, which quantifies the level of failure while adequately reflecting physical phenomena. We analyzed vibration data from all run-to-failure datasets to enhance the efficiency of learning by extracting common features related to failure from the vibration signals of all bearings. Following the data analysis, we present a skewness-based algorithm for automatic parameter selection in data preprocessing, along with techniques to overcome self-healing.

### 3.1. Data Analysis

Time domain signals used in some studies, such as EEMD [33,34], are influenced by phase, so even with the same data index, different samples may not share physical commonalities. However, frequency domain signals are not affected by a phase, meaning that even if the samples differ, the same data index corresponds to the same frequency, thus sharing significant physical commonalities. Therefore, we analyzed the frequency spectrum of the signals and observed that the natural frequency bandwidth varies for each system and bearing. Importantly, this frequency bandwidth is largely unrelated to the bearing or rotational frequency. Furthermore, in some cases, the tendencies of the frequency spectra change as the failure progresses. This implies that relying solely on a specific frequency information for feature extraction cannot capture sufficient characteristics of failure.

**Figure 3:**
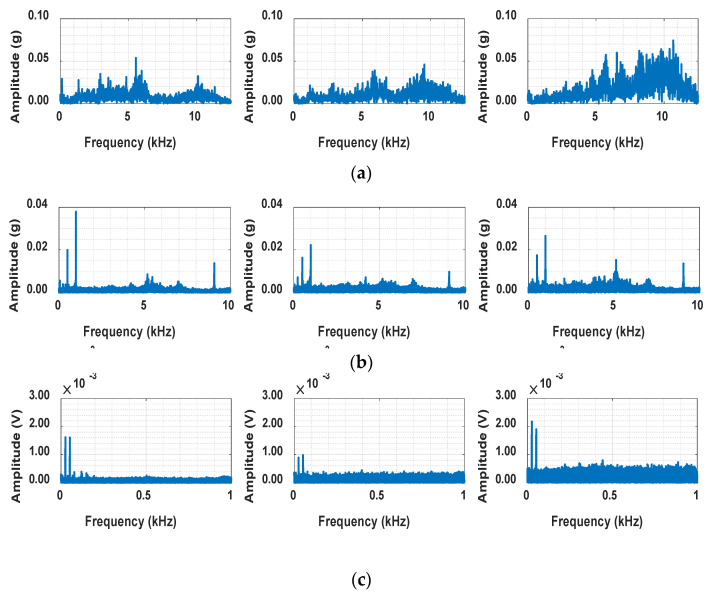
Energy increases at frequencies other than the natural frequency from healthy (left) to faulty (right): (**a**) PHM2012 2-1, (**b**) NASA IMS 2-1, and (**c**) the experimental setup.

We also discovered that as the failure progressed, there was a common increase in high-frequency energy in bandwidths where no energy had been manifested before [35,36,37]. For example, as observed in Figure 3c, with increasing fault severity, the energy in frequency bands that originally exhibited lower intensity—compared to the motor rotational frequencies at 17 Hz and 34 Hz—becomes more pronounced. This was also unrelated to the bearing’s rotational frequency. While the bearing enters the stage of fault, high-energy impacts excite the bearing’s natural frequency, and the high-frequency energy level starts to increase as the defect develops. This behavior was not specific to any particular bearing but was observed across almost all bearings. This phenomenon is known to occur because small cracks on the surface of the bearing generate small impulses, which in turn increase high frequencies.

### 3.2. Average PSD Construction

To utilize the noise increment phenomenon as a main feature of bearing degradation, the difference between the frequency spectrum in a healthy state and a current state was measured, employing the power spectral density (PSD) that prominently displays this phenomenon. PSD is the square of the Fourier transform (FT) that can diminish the energy at lower frequencies even further, thus accentuating the noise increment. An energy at frequency f can be calculated when there is a discrete signal x with a length of signal M.

(2) $$P\left[f\right]=\frac{1}{M}{\left(\sum_{n=0}^{K-1}x\left[n\right]\cdot e^{-\frac{i2\pi fn}{M}}\right)}^{2}$$

For practical implementation, it is first necessary to determine an average PSD, denoted as $\bar{P},$ for a comparison with the current PSD. This average PSD is the mean value of *N* PSDs from the initial operation of the bearing and using their mean, thereby extracting the average frequency trend in the healthy state.

(3) $${\bar{P}}_{N}\left[f\right]=\frac{1}{N}\sum_{i=1}^{N}P_{i}\left[f\right]$$

The selection of *N* focuses on not selecting excessively many samples, so *N* is continuously increased until the cross-entropy between ${\bar{P}}_{N-1}$ and ${\bar{P}}_{N}$ does not exceed a sufficiently small number, i.e., $10^{-6}$. Since cross-entropy quantifies the discrepancy between two vectors, it prevents excessive increases in data size even after a sufficient $\bar{P}$ has been obtained. The algorithm for constructing $\bar{P}$ is presented in Algorithm 1.

(4) $${C.E.}_{N}={\bar{P}}_{N-1}\log\left({\bar{P}}_{N}\right)$$

**Algorithm 1.**  $\bar{P}$ construction.Initialize *M*, *K*, *N* Initialize list $P_{i}$ while true do   for f from 0 to K−1 do   Compute $P\left[f\right]=\frac{1}{M}\sum_{0}^{K-1}{\left(x\left[n\right]e^{-\frac{i2\pi fn}{M}}\right)}^{2}$   end for   Add P to $P_{i}$   Compute ${\bar{P}}_{N}\left[f\right]=\frac{1}{N}\sum_{1}^{N}P_{i}\left[f\right]$   Compute $C.E._{N}={\bar{P}}_{N-1}\times log({\bar{P}}_{N})$   if $C.E._{N}<10^{-6}$ then   break   end if   Increment N end while

### 3.3. Skewness-Based Parameter Selection

After determining $\bar{P}$, it was smoothed and segmented to ensure a stable division of the entire frequency spectrum into equal intervals. This approach was adopted because the noise increment phenomenon is not confined to specific frequencies but occurs indiscriminately across the entire spectrum. Dividing the spectrum into sections enables the independent calculation of differences and the utilization of mean values. Furthermore, to ensure that the methodology was universally applicable to all bearings rather than specific ones, we proposed a skewness-based parameter selection algorithm to eliminate the use of arbitrary values when selecting parameters.

In a frequency spectrum, skewness quantifies the asymmetry in the distribution of amplitude values around the mean. A distribution with zero skewness is perfectly symmetric, meaning that amplitude values are evenly distributed around the mean. Positive skewness indicates a longer tail on the right side, suggesting the presence of relatively few high-amplitude values extending away from the mean. Conversely, negative skewness signifies a longer tail on the left side, indicating the presence of unusually low-amplitude values extending far below the mean. This measure aids in understanding the shape of the amplitude distribution and in detecting anomalies within the data.

(5) $$Skewness=\frac{n}{\left(n-1\right)\left(n-2\right)}\sum_{i=0}^{I-1}{\left(\frac{\left(x\left[i\right]-\mu \right)}{\sigma }\right)}^{3}$$

We observed that as the smoothing number increases, the skewness converges to a specific value. This occurs because the bandwidth affected by noise is removed from $\bar{P}$, leaving only the information of the main characteristic frequencies. At this point, smoothing was performed using a moving average with an overlap of 1. As the smoothing number increased one by one starting from 1, the skewness of the flattened $\bar{P}$ was calculated. When the difference between the skewness of the current and the previous smoothened $\bar{P}$ became less than $10^{-3}$, the increase in the smoothing number was stopped. The selected smoothing number was then used to smooth $\bar{P}$ with moving average, which was subsequently normalized to have a total sum of 1. This normalization is crucial because the self-healing phenomenon manifests in the increase–decrease–increase pattern of energy levels, thereby rendering the magnitude of changes insignificant.

Large positive skewness indicates that the mean is less than the median, suggesting that segments include peaks while containing substantial information from lower energy levels. Therefore, the segmentation parameter was chosen to maximize the sum of segments’ skewness. This concept is critical to clearly observe the noise increment phenomenon, which involves energy increases at frequencies that previously had little energy as the fault progresses. At this stage, the segmentation refers to dividing the entire PSD into equal intervals according to the segmentation number. To select the optimal segmentation number, it was increased incrementally from 1 up to the total number of data points. For each segmentation number, the sum of the skewness values of all equal intervals was calculated, and the segmentation number corresponding to the maximum sum of skewness was chosen. After determining the smoothing, normalization, and segmentation parameters, we processed $\bar{P}$ to create a “reference PSD”, i.e., $P^{*}$. The algorithm for selecting smoothing and averaging parameters is presented in Algorithm 2.
**Algorithm 2.** Skewness-Based Parameter Selection.Initialize μ, σ, and S while true do   Smooth $\bar{P}$ with smoothing number   Compute OldSkewness of $\bar{P}$   if $OldSkewness-NewSkewness<10^{-4}$ then   break   end if   Increment smoothing number    OldSkewness=NewSkewness end while Compute min-max normalization of P¯ for m from 0 to M−1 do   Segment $\bar{P}$ with m   Compute Skewness of all $\bar{P}$   Add sum(Skewness) to S end for Select segmentation by $max\left(S\right)$

### 3.4. Anti-Self-Healing Factor

**Figure 4:**
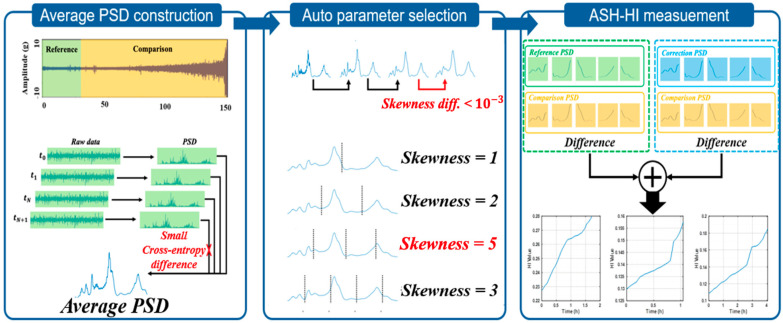
Schematic of ASH−HI feature extraction.

Once $P^{*}$ was established, the current PSD was preprocessed using the same parameters, and degradation was quantified based on the mean difference across identical segments. Furthermore, we proposed the ASH-HI, a method designed to mitigate the self-healing phenomenon not only by calculating the difference between $P^{*}$ and the current PSD but also by comparing the current PSD with the PSD recorded when the difference was at its highest. We defined this as the “correction PSD,” denoted as $P^{Co}$, which is continuously updated whenever a new maximum difference is detected. By continuously computing the difference between $P^{Co}$ and the current PSD and incorporating this difference with that between the current PSD and $P^{*}$, we derived the ASH-HI, as shown in Algorithm 3, which effectively prevents the misleading effects of the self-healing phenomenon. The procedure for deriving the ASH−HI is described in Figure 4.
**Algorithm 3.** ASH-HI Calculation.Define $P^{Co}$ Initialize difference, max difference for each current PSD do   Pre-process using the same parameters as for $P^{*}$  Compute mean difference across identical segments  if current difference>max difference then    Update $P^{Co}$    max difference=current difference  end if  Compute $ASH-HI=\left(P^{Co}-current\;PSD\;\right)+\left(P^{*}-current\;PSD\right)$ end for

## 4. Semi-Supervised Transfer Learning

We introduce a semi-supervised transfer learning methodology that enables the prediction of a target bearing’s RUL without the EOL information using the ASH-HI. Additionally, we present preprocessing techniques for RUL labeling based on the FPT and preprocessing of the ASH-HI, both of which were utilized to train the proposed deep learning model, consisting of an HI learner and a transformer.

### 4.1. RUL Labeling

**Figure 5:**
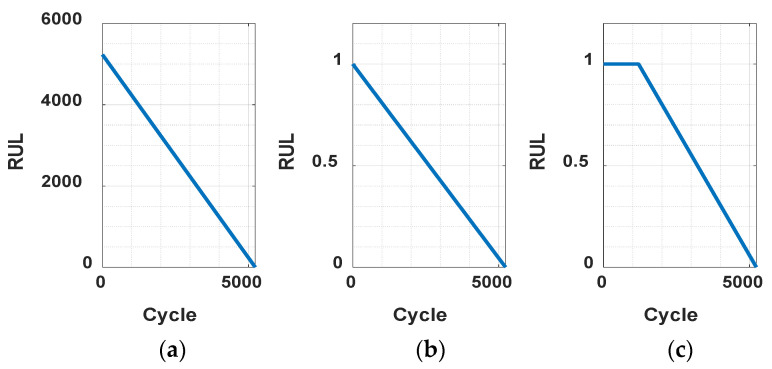
Normalization of RUL data: (**a**) raw RUL, (**b**) normalized RUL, and (**c**) normalized FPT RUL.

Bearings generally operate stably but then fail abruptly after a certain point, referred to as the FPT. Therefore, the FPT is defined as the point where failure begins in earnest, typically identified when an extracted feature deviates by more than three standard deviations from a predefined segment. Previous studies have labeled the bearing lifespan as 100% up until the FPT, as bearings operate stably until that point, and have modeled it as decreasing linearly thereafter [38,39,40]. In this research, the FPT was determined by identifying when the ASH-HI exceeds three standard deviations above the mean of the initial 200 ASH-HI values, since the EOL is unknown. The value was obtained from some references [38,39,40]. This value corresponds to approximately 15% of the total lifespan in the case of the shortest EOL in the RUL dataset used in this study, while it accounts for about 1% in the case of the longest EOL. Once the FPT was established, the time preceding this point was labeled as RUL 1, while the remaining time was assigned a linearly decreasing label from 1 to 0 until the EOL, as shown in Figure 5. This method provides a systematic and predictive approach to labeling the lifecycle of bearings based on their condition over time.

### 4.2. Baseline Architecture Model

Transformers [41] are well-suited for learning linguistic data, leveraging positional encoding, an embedding layer, and an attention mechanism. Word embedding is necessary because it has to be quantified to make the model understand human language, which has a large latent space. However, in field of RUL prediction, an embedding layer is not required since the HI has single- or low-dimensional data. Hence, we replaced the positional encoding and embedding layer with LSTM to learn the time-series patterns of the ASH-HI. Moreover, the original architecture of the transformer was designed for supervised learning, which leads supervised transfer learning. We added a layer predicting system HI at the end of the encoder to enable transfer learning without requiring the EOL information or the RUL from the target domain. The system HI consisted of 200 points from two ASH-HIs taken at the time of the FPT and the initial state. This size of (200, 2) data embedded the vibration characteristics related to performance degradation observed under bearing operation, and predicting it makes it possible to adapt the model with continuous labels, unlike in [19,20]. The predicted system HI and actual system HI were concatenated into a (200, 4) size and fed into a decoder to predict the bearing’s RUL. Meanwhile, since the system HI can vary across systems, making it challenging to train the model, we addressed this issue by concatenating three input data (200, 3) for the encoder: current, z-score, and min-max HI. The current HI denotes the raw ASH-HI at the prediction or reference time, while the z-score HI and the min-max HI represent the z-score and min-max normalized versions of the current HI, respectively. During normalization, the mean, standard deviation, maximum, and minimum values were computed based on the initial 200 time points rather than the current one. This approach fosters rapid learning for predicting diverse system HIs and aids in capturing the underlying characteristics of each mechanical system.

**Figure 6:**
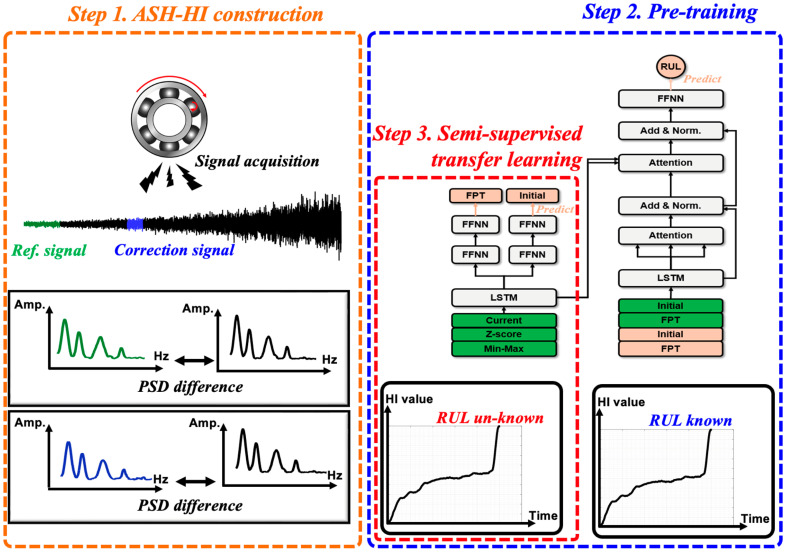
Pre-training and transfer learning schematic.

The proposed model learns to predict the target bearing’s RUL by implementing two steps, which are a pre-training step and a transfer learning step, as shown in Figure 6. In the pre-training phase, the model learns to predict RUL values using the current, the z-score, and the min-max value HIs containing EOL information, thereby reducing the prediction error of RUL. The learning rate was set to 0.001, and by training 100 times, a model was built that can predict RUL using the ASH-HI values. Furthermore, by predicting the system HI from the encoder’s output, the methodology simultaneously learns the characteristics of the system. In the transfer learning phase, the same training as in the pre-training phase is conducted once, and the encoder is alternately trained using a target domain dataset that does not include EOL information. In the transfer learning phase, by learning the system HI of the target domain from the encoder’s output, it achieves an effect similar to having learned RUL, even though it is not explicitly trained on it. During the inference phase, the estimation of the system HI is disabled, as only a prediction of RUL is needed.

## 5. Verification

In this section, to verify the excellence of the proposed methodology, we compare its performance with previous studies and investigate the training of data for the target bearing across multiple cases to ensure fair comparison and practical applicability. To verify the excellence of the proposed methodology, the HI measured trendability and robustness [42], while RUL measured mean squared error.

### 5.1. Comparison Studies

Two methodologies were adopted to apply transfer learning, allowing for a comparative analysis and validation of the proposed approach’s effectiveness. The first methodology follows a domain adaptation approach using a domain-invariant deep residual long short-term memory (LSTM) network. This approach estimates the RUL of the target bearing by making the deep learning model indistinguishable between the source and the target domains [43]. It extracts nine statistical features—RMS, variance, peak-to-peak, skewness, kurtosis, maximum, spectral skewness, spectral kurtosis, and wavelet energy—from vibration signals for pre-training and transfer learning without relying on RUL labels through domain adaptation. The second methodology does not utilize transfer learning but serves as a baseline to compare its performance against the more conventional supervised transfer learning-based RUL estimation technique [44]. This autoencoder-based deep learning methodology extracts the HI from vibration signals, which is then used to pre-train a convolutional neural network and an LSTM-based model. Subsequently, supervised transfer learning is applied to adapt the pre-trained model to the target bearing. Furthermore, to validate the superiority of the ASH-HI, an additional comparative analysis was conducted using an HI that does not incorporate the anti-self-healing factor. This comparison further highlights the effectiveness of the proposed ASH-HI approach in improving RUL prediction accuracy.

### 5.2. HI Verification


(6) $$Trendability=\frac{T\left(\sum_{k=1}^{T}x_{k}t_{k}\right)-\left(\sum_{k=1}^{T}x_{k}\right)\left(\sum_{k=1}^{T}t_{k}\right)}{\sqrt{\left[\left(T\sum_{k=1}^{T}x_{k}^{2}-{\left(\sum_{k=1}^{T}x_{k}\right)}^{2}\right)\right]\left[\left(T\sum_{k=1}^{T}t_{k}^{2}-{\left(\sum_{k=1}^{T}t_{k}\right)}^{2}\right)\right]}}$$

(7) $$Robustness=\frac{1}{T}\sum_{k=1}^{T}\exp\left(-\left|\frac{x_{k}-x_{k}^{T}}{x_{k}}\right|\right)$$

**Figure 7:**
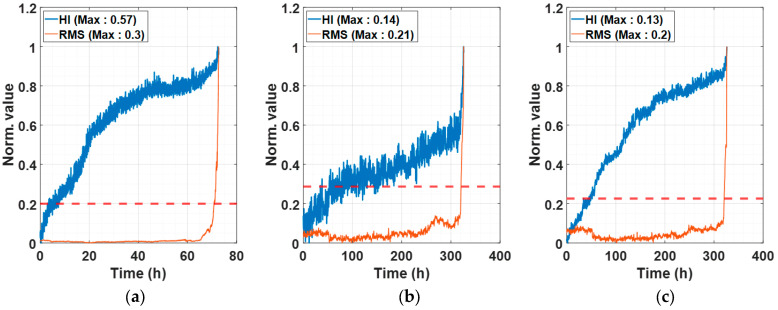

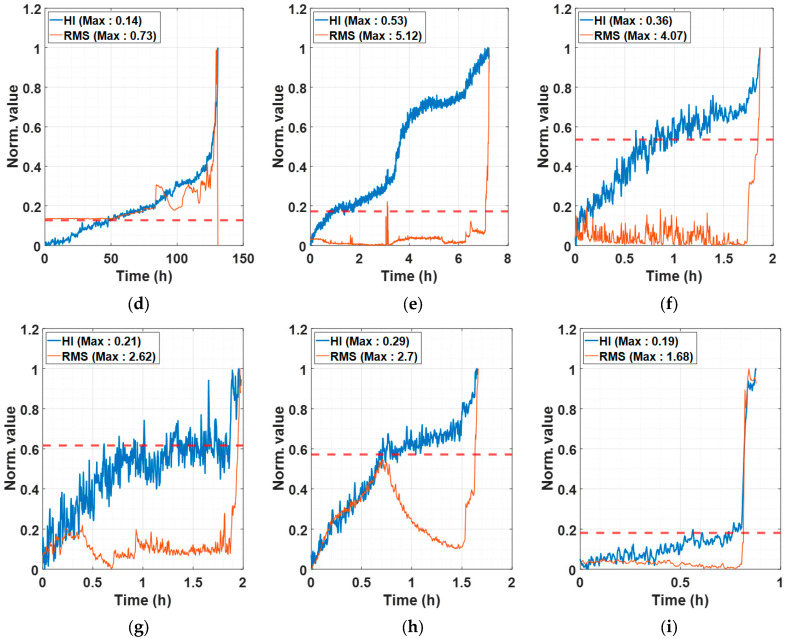
Normalized ASH-HI and RMS: (**a**) experimental setup, (**b**) NASA IMS 1-1, (**c**) NASA IMS 1-2, (**d**) NASA IMS 2, (**e**) PHM 2012 1-1, (**f**) PHM 2012 1-2, (**g**) PHM 2012 2-1, (**h**) PHM 2012 2-2, and (**i**) PHM 2012 3-1. The red dashed line indicates the FPT threshold.

Trendability [42] is evaluated by calculating the Pearson correlation coefficient between time and the HI, assessing whether HI values consistently increase over time. Robustness [42], on the other hand, is measured by comparing the difference between the HI’s trend component and the original HI, examining the volatility in the HI. Previous studies [42] often failed to achieve an average trendability above 0.9 and were limited to specific bearings. In contrast, the proposed ASH-HI demonstrates excellent trendability across all bearings, consistently surpassing an average of 0.9. Figure 7 illustrates the normalized ASH-HI and RMS for various bearings, where “Max” denotes the maximum value before normalization. As shown in Figure 7, the ASH-HIs steadily increase over time, effectively indicating the reduction in bearing life, even in cases where the self-healing phenomena cause RMS to recover dramatically. Notably, when using vibration signals without restricting the value range, the features at the EOL in Figure 7a–c,i are significantly larger compared to the others, making it challenging to predict progressive performance degradation. In contrast, the proposed ASH-HI progressively increases over time, enabling the quantification of performance degradation while also exhibiting a sharp increase as complete failure approaches. This characteristic aids in predicting the rapidly deteriorating condition of the bearing.

**Figure 8:**
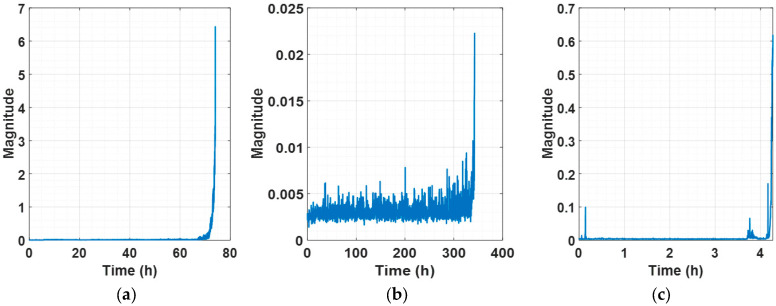
Extracted HIs time signals by [44]: (**a**) experimental setup, (**b**) IMS 1-1, and (**c**) PHM 2012 1-1.

**Figure 9:**
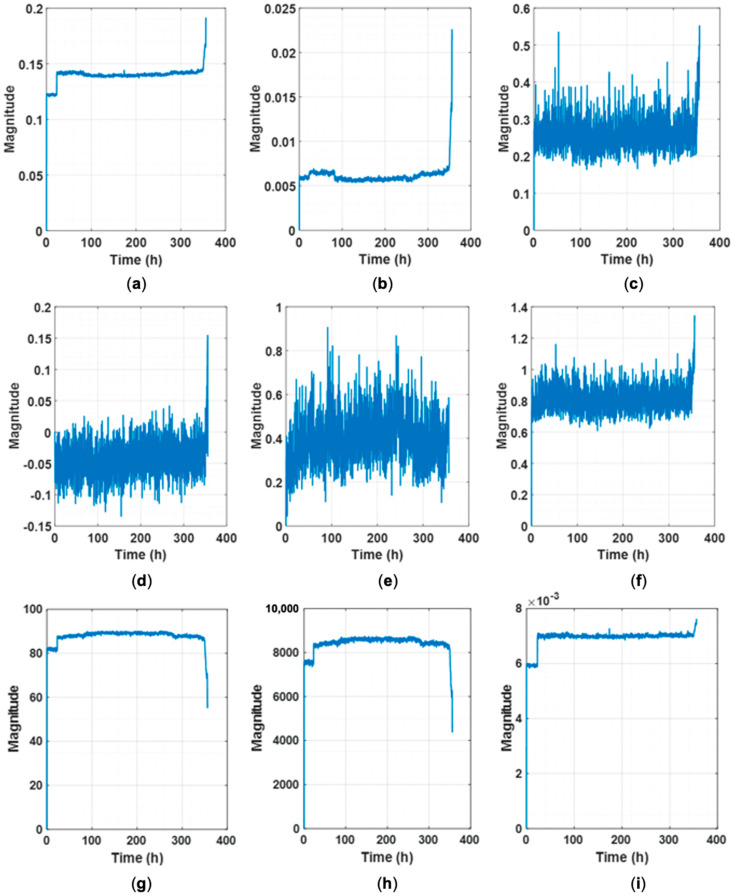
Extracted features time signals of IMS 1-1 by [43]: (**a**) RMS, (**b**) variance, (**c**) P2P, (**d**) skewness, (**e**) kurtosis, (**f**) maximum, (**g**) spectral skewness, (**h**) spectral kurtosis, and (**i**) wavelet energy.

**Figure 10:**
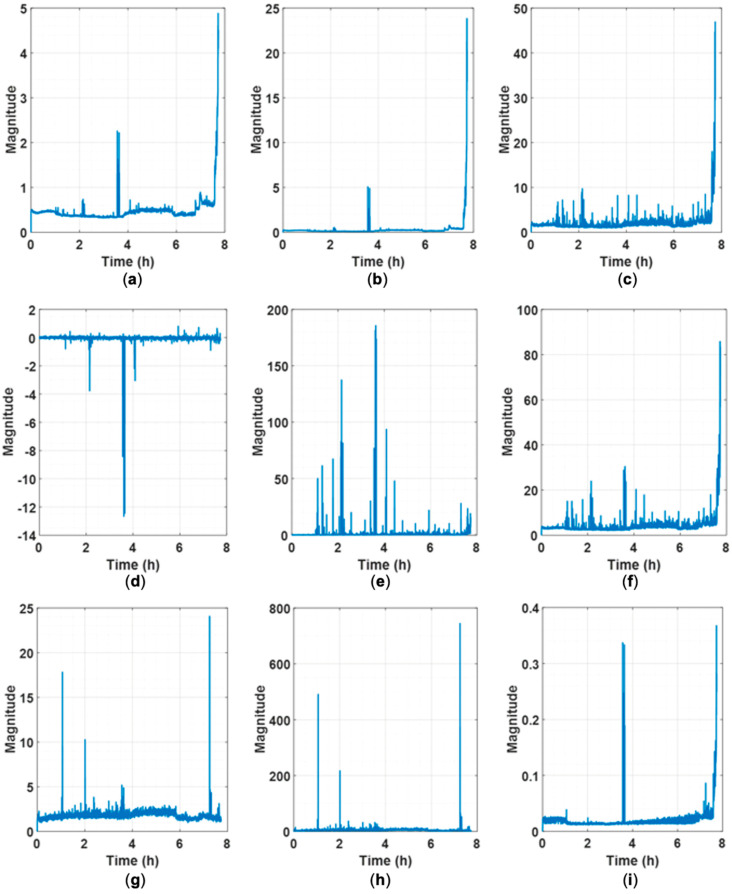
Extracted features time signals of PHM 1-1 by [43]: (**a**) RMS, (**b**) variance, (**c**) P2P, (**d**) skewness, (**e**) kurtosis, (**f**) maximum, (**g**) spectral skewness, (**h**) spectral kurtosis, and (**i**) wavelet energy.

The self-healing phenomenon is well demonstrated in Figure 7 at 100 h in (d), 6 h in (e), 0.7 h in (g), and 1.5 h in (h). Specifically, Figure 7d,h exhibit the most prominent self-healing effects, where RMS recovers significantly within an hour, yet ASH-HI continues to rise steadily over time. In addition, it is remarkable that the ASH-HI’s magnitude continues to grow even in the circumstance of a minor change in RMS, which represents the characteristics of vibration. Figure 7a–c,e,g,i demonstrate ASH-HI’s superiority by highlighting the performance degradation from the very early stage to the EOL. On the other hand, most of the features abruptly change at the very last moment of their RUL, as shown in Figure 8, Figure 9 and Figure 10. Table 3 demonstrates that such a sudden alarm ruins the trendability performance of features. In our experimental setup, two instances of self-healing behavior are observed at 61 and 69 h, as shown in Figure 7a. Despite these occurrences, the proposed HI robustly assesses that the bearing continues to deteriorate in performance. We also confirm some features, such as P2P, kurtosis, maximum, and spectral kurtosis of vibration, are sensitively changed over time, considering that the average of the robustness scores is 0.99, as shown in Table 4. These chaotic behaviors result in the poor robustness scores of the features and make them unreliable.

Another key advantage of the proposed ASH-HI is that it uses the average of differences between normalized PSDs, ensuring that its values do not exceed 1, unlike RMS. By confining input data within a consistent range, this approach provides a reliable method for assessing bearing life and contributes to improved model learning performance. Another notable observation is that just before the complete failure of a bearing, RMS sharply increases, signaling a rapid deterioration in fault severity. The proposed ASH-HI effectively responds to such rapid deterioration, providing a robust quantification of the bearing’s health status.

### 5.3. Mean Squared Error

Mean squared error calculates the average of the squared differences between the actual RUL and the predicted RUL to assess prediction accuracy. The performance of the proposed methodology was validated using cross-validation. In other words, among the ten datasets, the datasets excluding the target domain were intended to be used for the pre-training.

(8) $$MSE=\frac{1}{N}\sum_{n=1}^{N}{\left[RUL_{truth,n}-RUL_{pred,n}\right]}^{2}$$

To compare the RUL prediction performance without EOL information, simulations were conducted under the assumption that the proposed model has no access to the target bearing’s RUL data. After the pre-training, it only learns the target bearing’s system HI before the FPT and then it infers the RUL.

**Figure 11:**
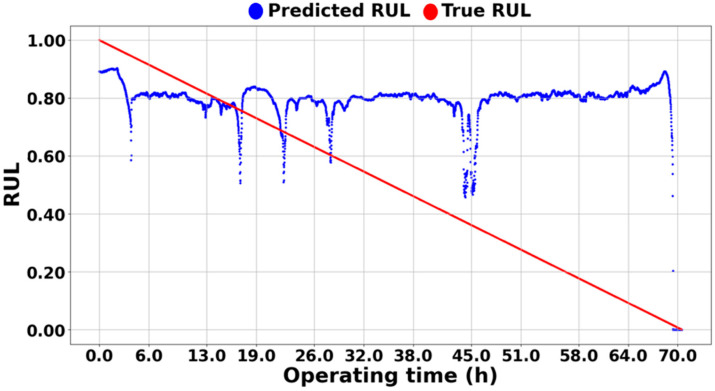
RUL prediction result of the experimental setup by [44].

**Figure 12:**
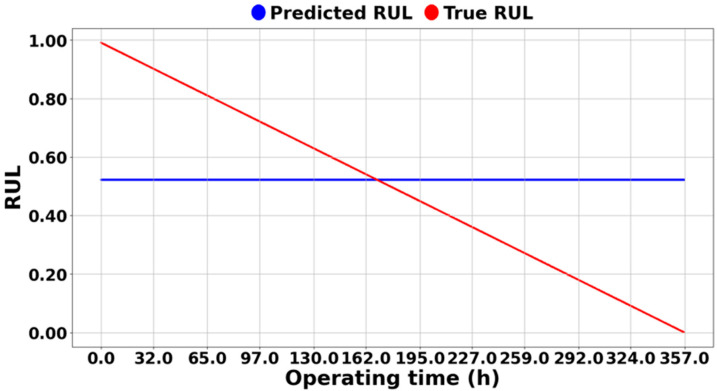
RUL prediction result of IMS 1-1 by [43].

Figure 11 shows the results of the RUL prediction of IMS 1-1 bearing data using the methodology in [43]. It can be seen that the predicted blue dashed line only predicts about 0.55 RUL, which indicates that the model has not learned any correlation between the input and the output data. The reason for such results is the significant differences in actual physical values of vibration between different systems across different datasets, which can be seen in the differences in values between Figure 9 and Figure 10. Figure 9a and Figure 10a represent the physical feature of the vibration magnitude using RMS. In the IMS dataset, an RMS of 0.2 indicates a very dangerous level, suggesting imminent bearing failure; however, this is not the case in the PHM dataset. If features are not extracted and learned within a consistent range, as in the case of Figure 11 or Figure 12, the prediction can only be learned to a level where it is completely impossible. Figure 12 shows the results predicted using the methodology in [44], and Figure 8 represents the extracted HIs. While this methodology appears to extract features within a consistent range (less than 1) from the open-source dataset, features extracted from the experiment setup exceed this range. From the model’s perspective, it is required to learn and predict values on a scale it has never encountered before. As a result, even with transfer learning, the prediction performance cannot be improved, leading to poor results, as shown in Figure 12. Consequently, if all datasets are combined for training, predictions are similarly impossible.

**Table 3:** Trendability results.

Dataset	Bearing #	ASH-HI	RMS	Variance	P2P	Skewness	Kurtosis	Maximum	Spectral Kurtosis	Wavelet Energy	[44]
PHM 2012	1-1	0.98	0.33	0.21	0.29	−0.01	0.02	0.28	0.04	0.01	−0.18
PHM 2012	1-2	0.94	0.22	0.25	0.03	−0.01	−0.02	0.04	−0.07	−0.08	0.50
PHM 2012	2-1	0.82	0.63	0.48	0.53	0.00	0.20	0.53	0.44	0.37	0.37
PHM 2012	2-2	0.95	0.58	0.47	0.53	0.26	0.28	0.55	0.11	−0.08	0.13
PHM 2012	3-1	0.84	0.34	0.35	0.34	−0.09	0.14	0.34	−0.09	−0.07	0.34
PHM 2012	3-2	0.97	0.27	0.21	0.28	0.00	0.05	0.27	−0.09	−0.04	0.20
NASA IMS	1-1	0.93	0.46	0.45	0.24	−0.28	−0.31	0.19	0.10	−0.01	0.31
NASA IMS	1-2	0.96	0.46	0.27	0.60	−0.15	0.37	0.56	−0.36	−0.36	−0.10
NASA IMS	2-1	0.83	0.46	0.45	0.24	−0.28	−0.31	0.19	0.10	−0.01	0.14
Experiment set	1	0.98	0.34	0.22	0.42	−0.25	−0.33	0.35	−0.47	−0.49	0.47

**Table 4:** Robustness results.

Dataset	Bearing #	ASH-HI	RMS	Variance	P2P	Skewness	Kurtosis	Maximum	Spectral Kurtosis	Wavelet Energy	[44]
PHM 2012	1-1	0.99	0.96	0.92	0.89	0.94	0.73	0.86	0.94	1.00	0.87
PHM 2012	1-2	0.99	0.93	0.88	0.49	0.94	0.25	0.52	0.93	0.99	0.77
PHM 2012	2-1	0.98	0.98	0.97	0.87	0.99	0.69	0.87	0.95	1.00	0.84
PHM 2012	2-2	0.99	0.99	0.98	0.94	0.97	0.89	0.94	0.95	1.00	0.30
PHM 2012	3-1	0.89	0.99	0.99	0.91	0.96	0.82	0.92	0.94	1.00	0.59
PHM 2012	3-2	0.98	0.99	0.99	0.87	0.99	0.69	0.89	0.95	1.00	0.85
NASA IMS	1-1	0.99	0.99	0.98	0.93	0.99	0.98	0.93	0.87	0.84	0.70
NASA IMS	1-2	0.99	099	0.97	0.89	0.98	0.76	0.85	0.99	0.99	0.67
NASA IMS	2-1	0.98	0.99	0.98	0.93	0.99	0.98	0.93	0.87	0.76	0.68
Experiment set	1	0.99	1.00	1.00	0.98	0.89	0.92	0.98	0.92	0.89	0.93

**Figure 13:**
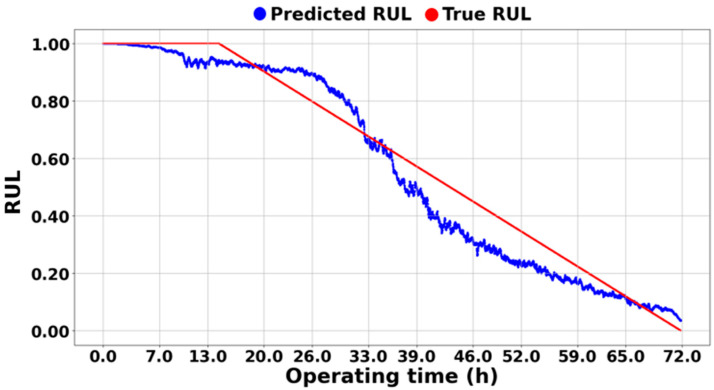
RUL prediction result of the experimental setup.

**Figure 14:**
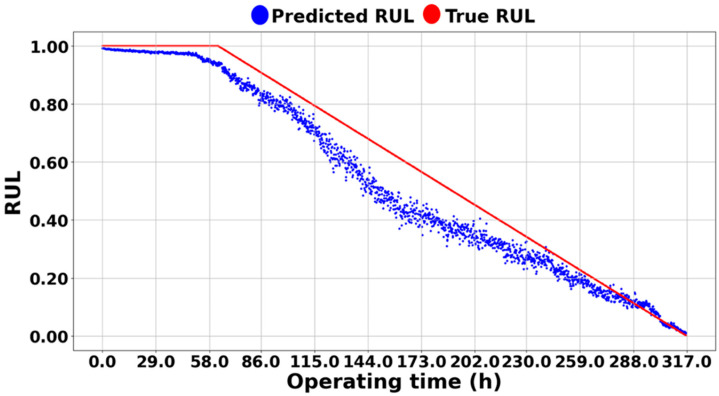
RUL prediction result of NASA IMS 1-1.

**Figure 15:**
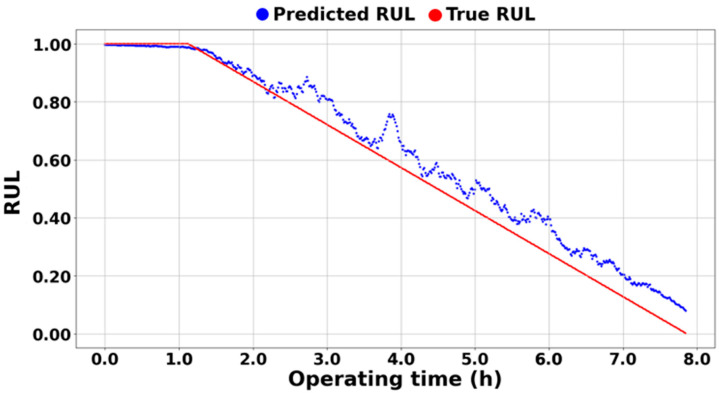
RUL prediction result of PHM 2012 1-1.

On the other hand, Figure 13, Figure 14 and Figure 15 demonstrate the excellence of the proposed RUL prediction methodology, showing that the difference between the blue predicted RUL values and the red actual values is not significant and that the trend is well followed. The ability to enhance RUL prediction performance using data from different systems is due not only to training with normalized data but also to the understanding of system characteristics through semi-supervised transfer learning in situations where RUL is not available. Additionally, it can be observed that the predictions do not deviate significantly and follow the trend line, which is due to all ASH-HIs having high robustness and consistently following the trend. Table 5 compares the MSE of the proposed algorithm with the other methodologies. As mentioned earlier, it was observed that the domain adaptation significantly underperformed, resulting in MSEs that were, on average, 35 times larger for [43] and 40 times larger for [44] compared to the proposed methodology.

**Figure 16:**
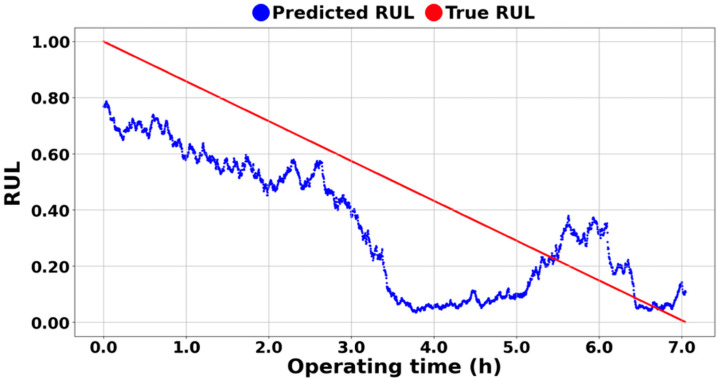
RUL prediction result of PHM 1-1 without the ASH-HI.

The superiority of the proposed ASH-HI is more pronounced when the model is trained using an HI that does not utilize the anti-self-healing factor. Figure 16 is an example showing the results for PHM 2012 1-1 without applying the ASH-HI. As shown in Figure 16, when using the deep learning algorithm, the self-healing phenomenon is mis-learned, causing the RUL to momentarily increase from 0.05 to 0.4, which can be observed at the 6 h mark. As previously mentioned, this does not indicate an actual improvement in the bearing’s health but rather an error caused by a temporary reduction in the measured vibration magnitude due to a physical phenomenon. The same self-healing phenomenon was also found at 6 h and 7 h of RMS, as shown in Figure 7e.

**Table 5:** MSE of RUL prediction for various deep learning algorithms. Grey highlighted figures indicate the best RUL prediction performance among various models and cases.

**Target Domain**	***w*/*o* Anti-Self-Healing**	**Proposed Model**	[43]	[44]
PHM 2012 1-1	0.0518	0.0091	0.2476	0.1161
PHM 2012 1-2	0.1131	0.0085	0.2400	0.1605
PHM 2012 2-1	0.0857	0.0097	0.2477	0.2717
PHM 2012 2-2	0.0486	0.0082	0.2670	0.5599
PHM 2012 3-1	0.0347	0.0040	0.2414	0.1489
PHM 2012 3-2	0.0648	0.0067	0.2450	0.3012
NASA IMS 1-1	0.0421	0.0011	0.2171	0.1374
NASA IMS 1-2	0.0276	0.0012	0.2353	0.2877
NASA IMS 2	0.0372	0.0023	0.2549	0.3305
Experiment	0.2439	0.0078	0.1628	0.1626

## 6. Conclusions

This study addresses two critical challenges in RUL prediction of slewing bearings: the self-healing phenomenon and the absence of EOL information in datasets. To overcome these issues, vibration data were analyzed, revealing that self-healing can obscure degradation trends by causing temporary reductions in the RMS of vibration signals. To counteract this effect, the correction PSD approach was introduced, ensuring a consistent and accurate assessment of degradation by comparing the current PSD with a dynamically updated reference.

Building on these insights, the ASH-HI feature extraction technique was developed to effectively track gradual degradation over time. The proposed ASH-HI demonstrated exceptional trendability and robustness, consistently exceeding 0.9 in performance metrics. Furthermore, to predict RUL without EOL labels, a transformer-based semi-supervised transfer learning framework was proposed. By incorporating the initial and FPT ASH-HI values, the model enhanced predictive accuracy for target bearings without requiring supervised RUL labels. This approach enabled adaptive transfer learning across different bearing types, systems, and data collection environments, marking a significant advancement in the field.

The proposed methodology was rigorously validated through a comparative analysis:

- It significantly outperformed traditional domain adaptation techniques, which exhibited MSEs 36 to 40 times higher.
- Supervised transfer learning using raw physical values suffered from overfitting, failing to capture the decreasing RUL trend accurately.
- Semi-supervised transfer learning with normalized features remained vulnerable to the self-healing phenomenon, leading to a performance degradation.
- The inclusion of an anti-self-healing factor in the health indicator was found to be crucial, as its absence resulted in substantially lower predictive accuracy, confirming its role in mitigating misleading effects caused by self-healing.

These findings highlight the effectiveness and robustness of the proposed methodology in addressing self-healing and unlabeled system challenges. By offering a scalable and practical approach to predictive maintenance, this study contributes to advancing real-world RUL estimation frameworks, improving reliability in industrial applications.

This methodology is not appropriate for bearings that change their rotating speed during the operation because the proposed ASH-HI estimates performance degradation by calculating the difference in the PSD between healthy and deteriorated vibration. The occurrence of a rotating speed change will cause an increment in the ASH-HI score even if the bearing is healthy. For future work, we will aim to make the ASH-HI applicable to bearings operating at variable speeds.

## Data Availability

The data that support the findings of this study are available from the corresponding author upon reasonable request.
